# Efficacy of radioactive iodine treatment of graves’ hyperthyroidism using a single calculated ^131^I dose

**DOI:** 10.1186/s40842-018-0071-6

**Published:** 2018-11-28

**Authors:** Ka Kit Wong, Barry L. Shulkin, Milton D. Gross, Anca M. Avram

**Affiliations:** 1 Nuclear Medicine/Radiology, University of Michigan, 1500 E. Medical Center Drive, Ann Arbor, MI 48109-5028 USA; 2 Nuclear Medicine/Radiology, St. Jude Children’s Research Hospital, Memphis, TN 38105 USA; 3 Nuclear Medicine Service, Department of Veterans Affairs Health System, Ann Arbor, MI 48105 USA

**Keywords:** Hyperthyroidism, Radioactive iodine, 131-I, Dosimetry, Graves’ disease

## Abstract

**Objective:**

To evaluate the success rate of therapeutic administration of a single calculated ^131^I activity for eliminating hyperthyroidism due to Graves’ disease.

**Methods and materials:**

Patients with Graves’ hyperthyroidism underwent pinhole thyroid imaging, 24-h radioactive iodine uptake (RAIU) measurements and clinical examination and received a calculated ^131^I activity of 0.2 mCi per estimated gram of thyroid tissue, adjusted for the 24-h RAIU. The goal of RAI treatment was to achieve hypothyroidism within 3–6 months of ^131^I administration. Response to RAI therapy was assessed at 7 weeks and 3 months by clinical and biochemical follow-up.

**Results:**

The study included 316 hyperthyroid patients with Graves’ disease (F238:M78, mean age 42.1 ± 16 y, 4–94). 179 patients (56.6%) had no prior therapeutic intervention (treatment-naive patients), whereas 6 patients had prior thyroid surgery, and 131 (41.5%) had been treated with anti-thyroid medications.

The mean estimated thyroid gland size was 50.2 g ± 18, range 15–100. Mean RAIU was 0.57 ± 0.17 (normal 0.07–0.30). RAI doses ranged from 5 to 70 mCi (mean dose = 18.1 mCi). Successful treatment of hyperthyroidism at our institution was obtained after a single therapeutic 131-I activity administration in 295 of 316 (93.3%) patients. Multivariate logistic regression analysis demonstrated that failure of ^131^I therapy was associated with previous PTU therapy (*p* <  0.001).

The mean response time after successful RAI therapy was 110.2 days, with cumulative response of 25% at 61 days, 50% by 84 days and 75% by 118 days after radioiodine administration. The mean time to respond for those on prior PTU medications was 297 days compared to 116 days for those on MMI and 109 days for those not previously treated with antithyroid medications. In patients with persistent hyperthyroidism, failure of RAI therapy was documented in 16 patients (76.2%) within (less than) one year after ^131^I administration and in 5 patients (23.8%) more than one year after initial therapy, considered late failure.

**Conclusion:**

Successful ^131^I therapy for Graves’ hyperthyroidism with a single calculated dose can be achieved in the majority (> 90%) of patients, adjusting for the thyroid size and 24 h uptake measurement.

## Introduction

Hyperthyroidism affects 0.5% of the population in the USA, with female preponderance (F5–10:M1) peaking between 40 and 60 years [1]. Radioactive iodine ^131^I (RAI) treatment is an effective definitive treatment of hyperthyroidism, used as a first line or second line treatment for over six decades, ^131^I being selectively concentrated by functioning thyroid tissue that subsequently is destroyed over weeks by beta-radiation [1–3]. ^131^I is a safe and well-tolerated treatment for hyperthyroidism due to Graves’ disease, multinodular goiter and autonomously hyperfunctioning nodule [4].

The goals and methods for delivery of RAI therapy vary between institutions with many published protocols. Of note is ongoing debate between use of high doses versus low doses of ^131^I [2] and whether the goals of treatment are immediate hypothyroidism, which is easier to manage with levothyroxine replacement, rather than long-term medical management of hyperthyroidism [5]. Some authors have proposed fixed doses of administered ^131^I, usually between 185 and 550 MBq (5–15 mCi), whilst others have adopted dosimetric approaches, based on calculations that take into account RAIU measurements and thyroid volume [1], selecting a dose of RAI therapy to attain a target absorbed dose to thyroid tissue [2].

Few investigators have evaluated the success rate of ^131^I therapy in eliminating hyperthyroidism, particularly with a single dose of radioactivity [6]. Our aim was to evaluate the success rate of RAI therapy in eliminating hyperthyroidism (goal of hypothyroidism and levothyroxine replacement), using a single dose of ^131^I with dosimetric approach and to determine any factors contributing to failure or prolonged response times.

## Methods and materials

### Patients

We reviewed clinical records and collected data on consecutive hyperthyroid patients referred between 1990 and 1998 to the University of Michigan Nuclear Medicine Therapy Clinic for radioactive iodine (RAI) thyroid gland ablation procedure for definitive treatment of hyperthyroidism. This time period of data collection reflects when the ^131^I RAI therapy protocol was initially developed, although the methodology is essentially unchanged from our contemporary practice. Approval for retrospective analysis of clinical and laboratory information with waiver of consent was obtained from the Internal Review Board.

Thyrotoxicosis was diagnosed on the basis of elevated free T4 (FT4) and/or total T3 (TT3) values and TSH suppressed to < 0.01 mIU/L. Subclinical thyrotoxicosis was diagnosed as normal FT4 and TT3 values with TSH suppressed between 0.3 to < 0.01 mIU/L on more than one occasion over several months of observation. The etiology of hyperthyroidism due to Graves’ disease was established by the combination of characteristic clinical features, laboratory data and results of thyroid imaging. Graves’ disease was diagnosed based on clinical examination; diffuse bilateral thyroid gland enlargement without nodules, either with or without typical findings of Graves’ eye disease and the presence of thyroid stimulating immunoglobulin assays (TSIS). When the history and clinical examination were uncertain, ^99m^Tc-pertechnetate thyroid pinhole imaging demonstrating a diffuse increased radiotracer activity throughout the thyroid gland, without focal nodularity, was considered an imaging pattern characteristic of Graves’ disease.

Patients on antithyroid medication had it held for at least 7 days prior to RAI therapy. In preparation for RAI therapy patients underwent clinical examination, ^99m^Tc-pertechnetate thyroid pinhole imaging, and RAIU uptake measurements at 24 h. Clinical evaluation included neck palpation to estimate thyroid gland size. Radioactive iodine uptake (RAIU) was measured after an oral 6–9 μCi dose of ^131^I with anterior neck counts measured with a probe placed at 25 cm from the patient at 24 h post administration; comparison was made with a daily standard corrected for background with a lead shield placed in front of the neck of the patient. 24 h RAIU was variable, but generally elevated (> 30% at 24 h).

We used a therapeutic ^131^I activity calculated to deliver a dose of 0.2 mCi per gram of estimated thyroid tissue at 24 h by the formula:

$$ {}^{131}\mathrm{l}\ \mathrm{activity}\ \left(\mathrm{mCi}\right)\ \mathrm{dose}=\frac{0.2\;\mathrm{mCi}\ \mathrm{per}\ \mathrm{gram}\ \mathrm{of}\ \mathrm{thyroid}\ \mathrm{tissue}\ \mathrm{x}\ \mathrm{target}\ \mathrm{thyroid}\ \mathrm{tissue}\ \mathrm{mass}}{\mathrm{RAIU}} $$

The desired dose to be retained in the thyroid is one that delivers 15,000–20,000 rad to the thyroid. This occurs when approximately 150–200 microCi/g is retained in the gland.

This dose is generally increased when: the gland is very large, there is evidence for very rapid turnover of ^131^I (higher 6 h than 24 h uptake, small gland), there has been prior anti-thyroid drug treatment, there is toxic nodular disease and when persons are severely toxic and a rapid response is desired (although a cooling off period is required).

Clinical follow-up was performed at 7 weeks and 3 months after ^131^I administration and included review of clinical symptomatology, physical examination and relevant laboratory data. Successful RAI therapy for hyperthyroidism was defined as absence of hyperthyroid symptoms and/or presence of symptoms of hypothyroidism; and normal to elevated TSH concentrations, subnormal to normal FT4 levels and TT3 levels, a pattern consistent with onset of hypothyroidism, but prior to the development of biochemical hypothyroidism (elevated TSH). Thyroid hormone replacement therapy was initiated when laboratory evidence of hypothyroidism was established. Treatment failure was defined as post-RAI therapy persistent laboratory evidence of hyperthyroidism after discontinuation of thyroid hormone replacement therapy.

### Statistical analysis

Continuous ordinal data is presented as mean ± SD. Failure rates are presented as percentages of the total within each category. Student t-test was employed to compare parametric data while Mann-Whitney tests were used to compare non-parametric data. Frequency was analyzed with Chi-squared and Fisher’s exact test. Event analysis was performed using time series data, with an “event” defined as success after RAI treatment and the time in days after RAI treatment used to generate Kaplan-Meier curves for response times observed to achieve hypothyroidism. Wilcoxon (Breslow) test for equality of survivor (event) functions and Cox proportion hazards regression were used to assess co-factors to determine effect on odds ratio of successful ablation. Statistical analysis was performed in STATA version 11 (Stata Corp, College Station, TX). Significance of findings was established at *p* <  0.05.

## Results

Over a nine-year interval 316 patients (mean age 42.1 ± 16 y, range 4 to 94) were treated with RAI for hyperthyroidism due to Graves’ disease. The group was comprised of 238 (75.3%) women and 78 (24.6%) men, including 20 pediatric patients < 18 years old at diagnosis. Patients with hyperthyroidism due to toxic multi- or uni-nodular goiter were excluded. Review of treatment history for hyperthyroidism prior to ^131^I administration revealed: 179 patients (56.6%) had no prior therapeutic intervention (treatment-naive patients). The remaining 137 (43.4%) patients had received prior treatment for hyperthyroidism as follows: medical treatment with anti-thyroid medications in 131 (41.5%) patients, propylthiouracil (PTU) in 80 (25.3%) patients, methimazole (MMI) in 37 (11.7%) patients, both PTU and MMI in 14 (4.4%) patients, and thyroid surgery in 6 (1.9%) patients, either lobectomy or partial thyroidectomy. In one patient partial thyroidectomy was performed followed by PTU therapy for persistent hyperthyroidism, while in a second patient MMI was given also after ineffective partial thyroidectomy. Table 1.

**Table 1:** Patient demographics

Characteristics	Number of patients (%)
Age	42.1 ± 16 y, 4–94^a^
Pediatric pts. < 18 y	20 (6.3)
Adult pts. ≥ 18 y	296 (93.7)
Total	316
Gender	
Female	238 (75.3)
Male	78 (24.6)
Etiology of hyperthyroidism	
Graves’ disease	316 (100%)
History of prior treatment	
None	179 (56.6)
Anti-thyroid medications	
Propylthiouracil	80 (25.3)
Methimazole	37 (11.7)
Both (PTU and MMI)	14 (4.4)
Total	137 (43.4)
Surgery	
Yes	6 (1.9)
Interval between diagnosis and radioiodine treatment	325.9 d, 0–6818
Radioiodine treatment	
Size of gland (g)	50.2 ± 18.1, 15–100^a^
24-h RAIU	0.57 ± 0.17
^131^I dose administered (mCi)	18.1 ± 6.8, 5–70^a^
Dosing factor (mCi per gram)	0.21 ± 0.14
Outcomes at First RAI Therapy	316
Success	295 (93.3)
Failure	21 (6.6)
Failure (needed 2 RAI doses)	18 (5.7)
Failure (needed thyroidectomy)	1 (< 0.3)
Failure (needed 3 RAI doses)	2 (< 0.7)
Interval between radioiodine treatment and hypothyroidism	97.3 d ± 78.4

^a^presented as mean ± SD, range

The mean time between diagnosis of hyperthyroidism and referral for RAI therapy was 325.9 days, with 78.2% of patients referred for RAI treatment within 1 year, and the longest interval being 18 years. The mean thyroid gland size was 50.2 g ± 18, range 15–100. The mean 24-h RAIU was 0.57 ± 0.17 (normal 0.07–0.30). The mean calculated RAI activity was 18.1 ± 6.8 mCi. The mean actual dosing factor delivered was 0.21 ± 0.14, matching the usual intended dosing factor of 0.2 mCi per gram in our clinical practice.

Hyperthyroidism was successfully treated in all patients eventually and after confirming hypothyroidism they were commenced on levothyroxine replacement (mean dose 109 mcg per day) during follow-up, the longest follow-up interval being 2.7 years. No patients were found to be euthyroid without hormone replacement after RAI therapy. We excluded 7 patients from analysis of our institutional failure rate, whom had a history of prior RAI treatment, these patients being treated at outside institutions by different methods based on fixed ^131^I doses without calculations based on goiter size and RAIU. Six of these 7 patients were successfully treated by RAI therapy at our center, the last needing one more RAI dose administration to achieve hypothyroidism.

The intended goal of achieving hypothyroidism with RAI treatment using a single dose administration occurred in 295 of 316 (93.3%) patients. There were 21 patients who failed their first RAI therapy; of these 18 patients subsequently developed hypothyroidism after receiving a 2nd RAI therapy, 2 patients required a total of 3 RAI treatments to achieve hypothyroidism, and 1 underwent thyroidectomy with subsequent cure of their hyperthyroidism, due to patient decision after reconsidering their treatment options.

Among 78 men, 69 (88%) were successfully treated with a single dose of RAI treatment and 9 had persistent hyperthyroidism. Among 238 women, 226 (95%) were successfully treated and 12 had persistent hyperthyroidism. The RAI therapy failures in men were significantly higher than in women (12 vs. 5%, *P* = 0.046). Among patients with failed RAI therapy and persistent hyperthyroidism, 17 patients (80.9%) were previously treated with antithyroid medications, MMI, PTU or both, while only 3 patients (14.2%) were treatment-naive (*p* <  0.001). By univariate analysis successful treatment was more likely with smaller mean thyroid size (49.3 g versus 62.1 g, *P* = 0.0017) (see Table 2).

**Table 2:** Co-factors between cohorts of success and failure of first RAI therapy for hyperthyroidism

Variable	Success (*n* = 295)	Failure (*n* = 21)	*P* value
Age (y) at time of diagnosis	41.9	45.5	NS (*P* = 0.32)
Gender (F:M)	226:69	12:9	(*P* = 0.046)
No prior treatment	176 (59.7%)	3 (14.2%)	(*P* < 0.001)
Prior antithyroid medication	114 (38.6%)	17 (80.9%)	(*P* < 0.001)
Methimazole	*n* = 35	*n* = 2	(*P* < 0.001)
Propylthiouracil	*n* = 79	*n* = 15	(*P* < 0.001)
Interval from diagnosis to treatment (d)	329.6	289.6	(*P* = 0.024)
Size of thyroid gland (grams)	49.3	62.1	(*P* = 0.0017)
24-h RAIU uptake	0.56	0.62	NS (*P* = 0.235)
Dose of RAI ^131^I administered (mCi)	18.1	19.9	NS (*P* = 0.193)
Dosing factor (mCi per gram)	0.21	0.19	NS (*P* = 0.259)
Weight (lbs)	150.1	136.8	NS (*P* = 0.050)
Thyrotropin (TSH) (normal 0.3–5.5 mIU/L)	0.13	0.010	NS (*P* = 0.948)
Total tri-iodothyronine (TT3) (normal 95–170 pg/mL)	328	415	NS (*P* = 0.139)
Free thyroxine (FT4) (normal 0.73–1.79 ng/dL)	3.2	3.8	(*P* = 0.026)
Race: caucasian	212 (71.8%)	11 (52.5%)	NS (*P* = 0.195)

Multivariate logistic regression analysis demonstrated that failure of ^131^I therapy was associated with previous PTU therapy (p 0.004) but not with MMI therapy, dose of ^131^I therapy, RAIU values before therapy or dose of ^131^I administered. Gender and size were no longer significant by multivariate analysis.

**Fig. 1:**
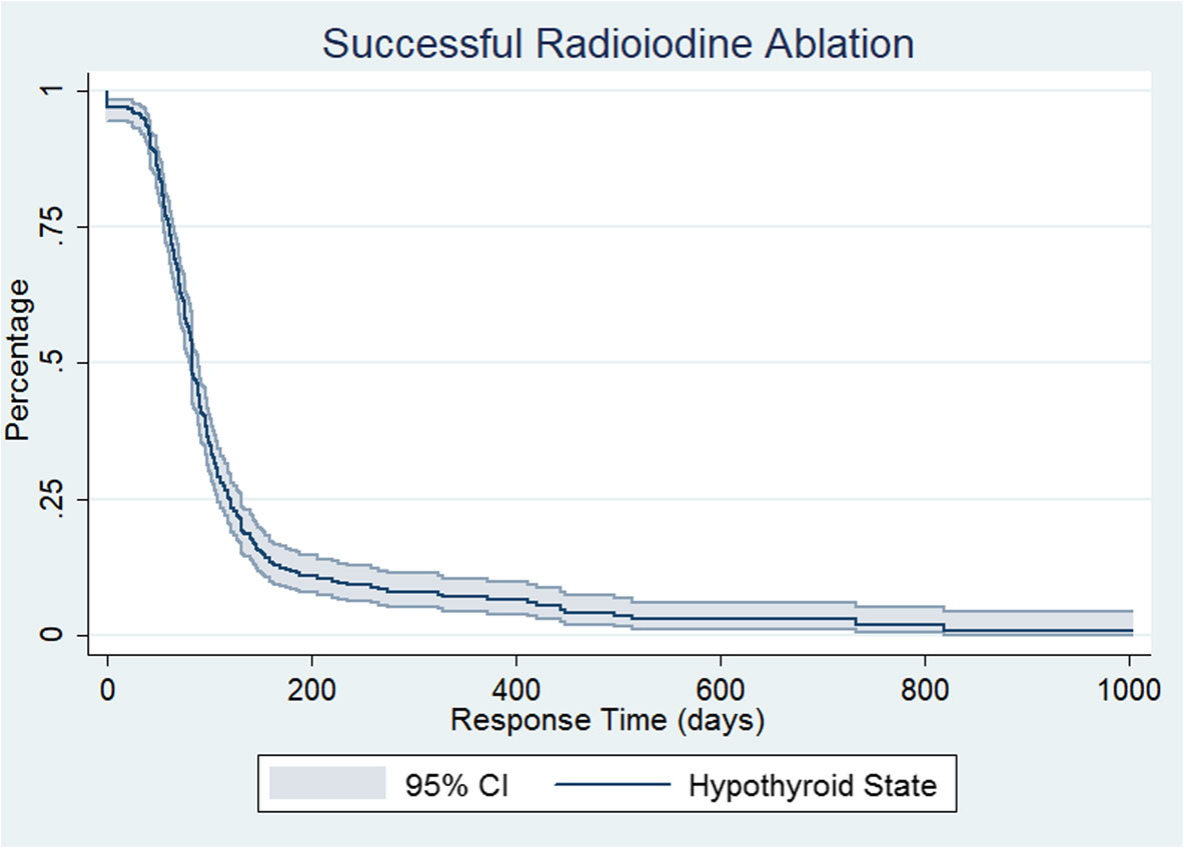
Kaplan-Meier response curve for successful RAI therapy

**Fig. 2:**
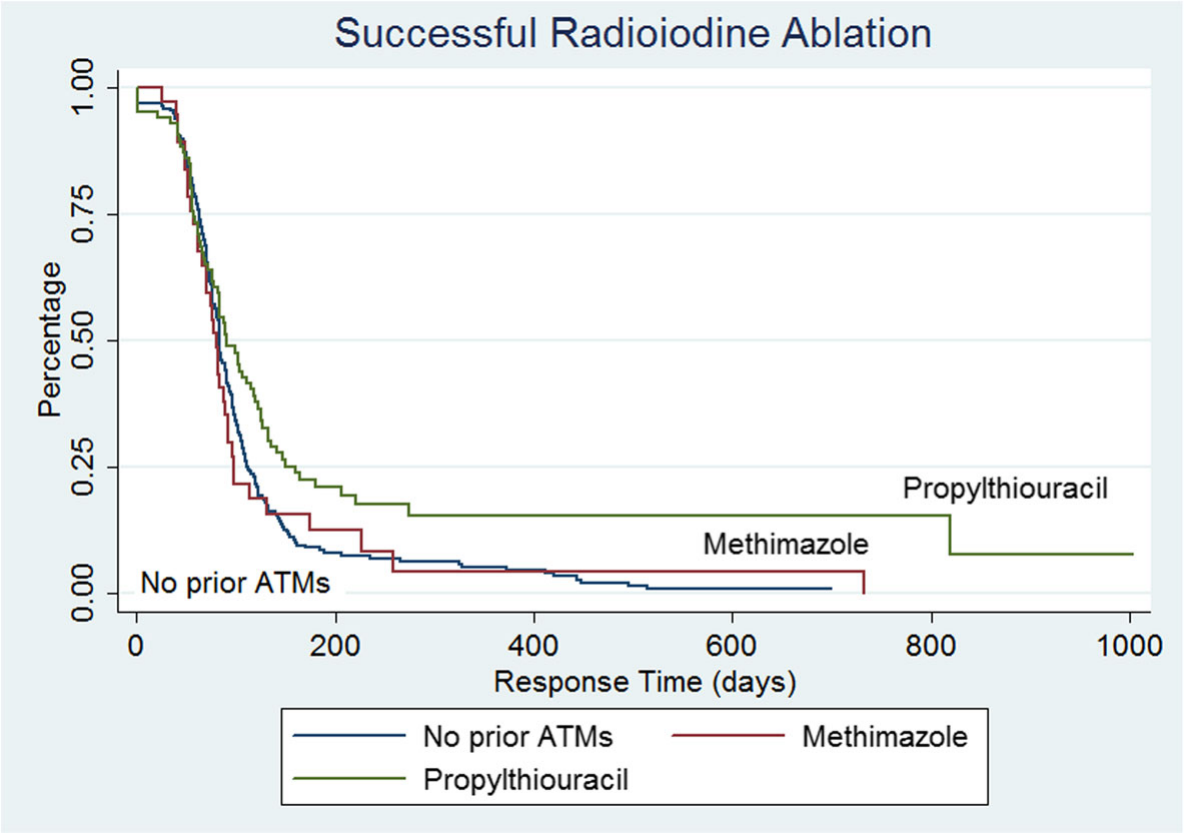
Kaplan-Meier response curve for successful RAI therapy by prior treatment history

**Fig. 3:**
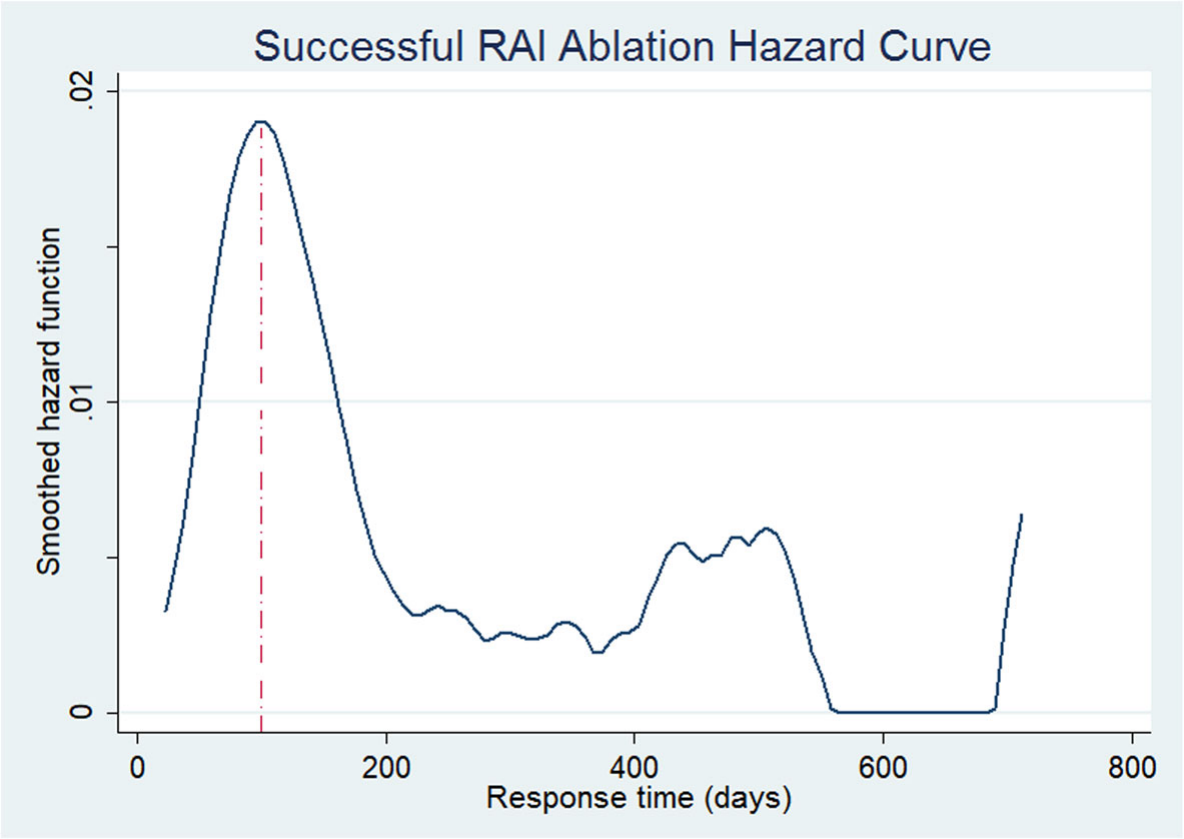
Hazard curve for successful RAI therapy

In 295 patients with successful ablation after their first RAI treatment, the mean time to response defined as development of hypothyroidism and initiation of levothyroxine replacement was 97.3 days ±78.4 range 1 to 733 days. Using event analysis of the successful ^131^I treatment (where an event was defined as time to successful treatment), Kaplan-Meier analysis of all treated patients demonstrated an exponential function and the mean response time after successful RAI ablation was 110.2 days, with cumulative response rate of 25% at 61 days, 50% by 84 days and 75% by 118 days (Fig. 1). The mean time to respond for those on prior PTU medications was 297 days compared to 116 days for those on MMI and 109 days for those not previously treated with antithyroid medications (Fig. 2). Regarding early versus late responders, there were approximately 91% responding by 200 days post RAI (~ 6 months) with ~ 9% late-responders (> 6 months). This represented the greatest hazard ratio for success occurred at 100 days (Fig. 3). In 21 patients with persistent hyperthyroidism, failure of RAI therapy was documented in 16 patients (76.2%) within (less than) one year after ^131^I administration and in 5 patients (23.8%) more than one year after initial therapy, considered late failure.

## Discussion

Many endocrinologists prefer an ablative dose of radioactive iodine to treat Graves’ hyperthyroidism. Doses of ^131^I in the range of 0.15–0.2 mCi per gram of thyroid tissue produce quicker resolution of hyperthyroidism than smaller dose therapies, lessening morbidity related to hyperthyroidism [7]. Although, higher doses of ^131^I are associated with higher success rates of RAI therapy this must be balanced with the need to avoid unnecessary absorbed radiation dose to the patients, family members and members of the general public, in accordance with the As Low As Reasonably Achievable (ALARA) principle. Conversely, treatment failures are associated with additional RAI treatments and dose exposure. American Thyroid Association (ATA) guidelines endorse the goal of hypothyroidism which can be achieved with simple fixed versus dosimetric approaches, although there is a recommendation for calculated doses > 150 microCi per gram of thyroid tissue [1].

We adopted goals of ending hyperthyroidism within 3–6 months to limit disability and to reduce the costs of multiple therapies. Our treatment goal is hypothyroidism, which is easier to manage with levothyroxine replacement, rather than long-term medical management of hyperthyroidism [5]. This is due to the inability to calculate dose with the aim to achieve euthyroidism, the progression to hypothyroidism even after a period of euthyroidism and the higher risk of recurrent hyperthyroidism which may occur if hypothyroidism is not achieved [5, 8]. By using prescriptions of 0.2 mCi per gram of thyroid tissue calculated from clinical estimates of thyroid volume, and ^131^I uptake at 1 day, we show that persistence of hyperthyroidism was 7% overall and that these failures were largely associated with prior propylthiouracil (PTU) therapy. No significant differences were found in outcomes related to RAIU or dose of radioactivity. By univariate analysis age, size and gender were significant factors influencing success rate of RAI therapy, however by multivariate analysis these factors became non-significant, possibly due to older patients receiving antithyroid medications. Furthermore, it is very likely that the patients with increased time interval were managed on anti-thyroid medications, and during this time the goiter enlarged.

In a nine year prospective study of ^131^I versus antithyroid medications, Chen and co-workers treated 209 patients with RAI therapy (178 single, 26 twice and 5 three times), using calculated doses ranging between 185–444 MBq [6]. Patients were not pretreated with antithyroid medications. Radioiodine was safe with no cases of thyroid storm or leukemia observed. Those treated twice or three times needed 263 ± 123 MBq. There was a 100% cumulative permanent hypothyroid rate at 100 months, with just over 24.4% conversions to hypothyroidism at 6 months and 62.2% achieved by 2 years. Of note similar to our findings higher gland weight was associated with longer time to cure (*P* = 0.001). The authors concluded that low-dose radioiodine has better cure rates than antithyroid medications, and lower relapse rates, at the expense of higher percentages of hypothyroid patients.

In one randomized trial of fixed versus calculated doses it was reported that fixed doses had success rate of 71% versus calculated doses of only 58%, however the success rate of fixed doses decreased with higher thyroid volumes (> 75 g) to only 25%. Whereas, in these large thyroid glands the calculated doses had 45% success rate [2]. Traino and colleagues looked at 3 different calculated doses with 400 Gray (Gy) delivered to the thyroid gland having the highest cure rate of 97% [9]. In a study of 84 patients treated with RAI therapy followed at 4–5 months, 14 were euthyroid, 49 hypothyroid and 21 hyperthyroid. No single factor predicted early hypothyroidism in this study [10]. In a study of 72 patients treated with calculated dose a significant difference in outcomes between low versus high-dose was noted [11]. A meta-analysis was inconclusive for superior outcomes or efficacy between fixed versus calculated doses [12].

The outcomes of 119 Graves’ disease patients were reported treated with a dosimetric protocol between 2003 and 2008, along with simulations of other protocols, followed for 12 months after treatment. A group receiving 120–200 Gray (Gy) radiation dose to the thyroid was prescribed, leading to a cure of hyperthyroidism with a single radioiodine administration in 53% of patients. When a higher radiation dose to the target (200–250 Gy) was prescribed this outcome raised up to 89% although the administered activities were still lower, as a rule, than the most commonly employed fixed activities (400–600 MBq). The authors found that there existed a high level of individual dose optimization, particularly when compared to simplified methods [13]. In a study of 59 patients the dosimetric method was used for diffuse goiter and nodular goiter. The protocols, which have not taken into account the thyroid mass, multi pre-therapeutic ^131^I uptakes and the effective half-life of ^131^I of the individual patient, showed a higher degree of deviation from the required thyroid dose. According to the results for diffuse goiter and nodular goiter, respectively, 28 and 54% patients were under-dosed and 72 and 46% patients were over-dosed [14].

A study of patients randomized into two treatment groups, one receiving 60 Gy and the other receiving 90 Gy thyroid tissue absorbed dose of radioiodine. At 6 months, five patients in the 90 Gy group were hypothyroid, compared to two patients in the 60 Gy group (*P* = 0.246). Overall at 6 months, non-responders to low-dose therapy had a significantly larger thyroid gland mass (respective means: 35.9 ml vs 21.9 ml). Where low-dose radioiodine treatment of Graves’ disease is considered, a dose of 60 Gy will yield a 39% response rate at 6 months while minimizing early hypothyroidism. No significant advantage in response rate is gained by using a dose of 90 Gy [15].

The use of antithyroid medications and the effect on RAI therapy remains controversial, although with a number of different protocols were tested taking into account history of prior antithyroid medication use [16]. We found that PTU, but not MMI to be associated with delayed response time. There is a suggestion that PTU may cause increased resistance of thyroid tissue to radiation effect and subsequent successful response rates [17–19]. Others have not reported this to be an issue with MMI or carbimazole [20, 21]. In a meta-analysis of 14 RCTs with 1269 patients [7], the risk of antithyroid medications on unsuccessful RAI therapy was 1.28, CI 1.07–1.52 (*p* = 0.004) compared to no antithyroid medications. There included MMI (6 trials), PTU (4 trials), carbimazole (3 trials) and one comparing PTU with MMI. The effect on achieving hypothyroidism was independent of antithyroid drug choice [7].

One of the limitations of our study was that we were unable to report on the adverse effects attributable to the ^131^I administration as this data was not consistently recorded in the medical record. Our data collection was from the time period when the protocol for the calculation of ^131^I doses was initially developed. However, we consider the results of this study applicable to our contemporary practice, as the protocol is largely unchanged from when it was initially developed. The only change has been the introduction of a 1 week low-iodine diet preparation around 3 years ago which could affect the uptake measurements though not the efficacy of ^131^I administration. Another limitation of this study are we were unable to evaluate the impact of TSIS on treatment failure. Finally, the mean time to response was calculated based on follow-up result of hypothyroidism which may have underestimated the true time to hypothyroidism. However, since patients called with symptoms of hypothyroidism and were brought in the discrepancy in our measured time to response and actual development of hypothyroidism was minimal.

## Conclusion

Using calculations of dose of RAI based upon thyroid gland size and correcting for radioiodine uptake, successful treatment with a single dose of RAI can be achieved with a 7% first therapy failure rate, with few patients requiring additional doses of RAI.

## Abbreviations

(MMI): methimazole
(PTU): propylthiouracil
(RAI): radioactive iodine
(RAIU): radioactive iodine uptake
